# Hemoperitoneum associated with uterine fibroids: A case report

**DOI:** 10.1097/MD.0000000000024024

**Published:** 2021-03-12

**Authors:** Atsushi Daimon, Tomohito Tanaka, Yuhei Kogata, Yoshimichi Tanaka, Daisuke Fujita, Masahide Ohmichi

**Affiliations:** Department of Obstetrics and Gynecology, Osaka Medical College, 2–7 Daigakumachi, Takatsuki, Osaka, Japan.

**Keywords:** case report, computerized tomography, hemoperitoneum, uterine fibroid

## Abstract

**Introduction:**

Uterine fibroids, which are common benign tumors, rarely cause acute complications. We herein report a case of hemoperitoneum associated with uterine fibroid that could be diagnosed preoperatively with contrast-enhanced computerized tomography (CT).

**Patient concerns:**

A 48-year-old woman with uterine fibroid developed extremely severe lower abdominal pain on the first day of her menstrual period.

**Diagnosis:**

Ultrasonography and contrast-enhanced CT revealed a uterine fibroid and extravasation from the dilated vessels of the uterine fibroid.

**Intervention:**

Emergent abdominal hysterectomy was performed.

**Outcomes:**

The total amount of bleeding was 4,600 mL. Intraoperative blood salvage (1,357 mL), 6 units of red blood cells, 4 units of fresh frozen plasma, and 20 units of platelet concentrates were transfused. The postoperative course was uneventful. Pathological examination confirmed a benign uterine fibroid.

**Conclusion:**

CT could be useful to determine a diagnosis for bleeding from ruptured subserosal uterine fibroid.

## 1 Introduction

Uterine fibroids are the most common benign pelvic tumors in women.^[1]^ The typical symptoms are hypermenorrhea, prolonged menstrual bleeding, painful menses, bulk symptoms including bowel and bladder dysfunction and abdominal protrusion, infertility, and recurrent miscarriage.^[2]^ Hemoperitoneum of gynecological origin may occur in the context of various gynecological emergencies such as ectopic pregnancies or ruptured corpus luteal cysts.^[3]^ Hemoperitoneum associated with uterine fibroids is extremely rare^[4]^; and difficult to diagnosis preoperatively.

We herein report a rare case of hemoperitoneum associated with uterine fibroids that could be diagnosed preoperatively with contrast-enhanced computerized tomography (CT). In addition, a literature search was conducted regarding triggers, preoperative diagnosis, and treatment of this condition.

## 2 Case presentation

This study was approved by the Osaka Medical College Clinical Research Review Board, and the patient gave written informed consent for publication.

**Figure 1:**
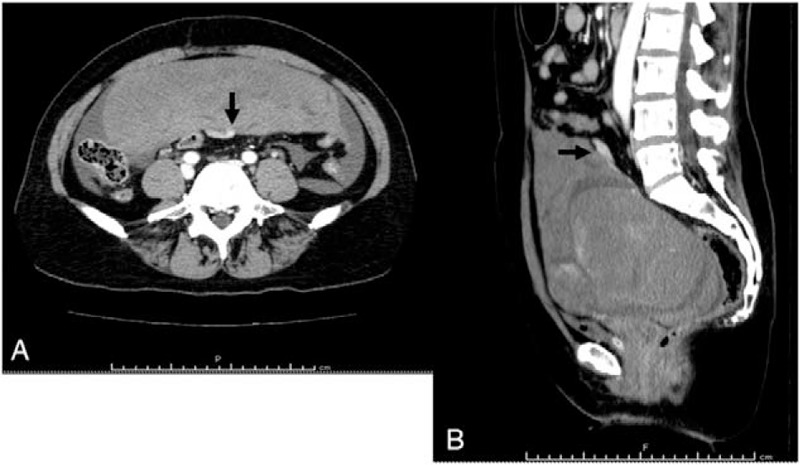
Abdominal contrast-enhanced computed tomography (CT) image. (A) Transverse section. (B) Sagittal section. A large amount of hemorrhagic ascites was noted, as was a uterine fibroid. The black arrow indicates extravasation from the dilated vessel on the dorsal side of the uterine fibroid.

**Figure 2:**
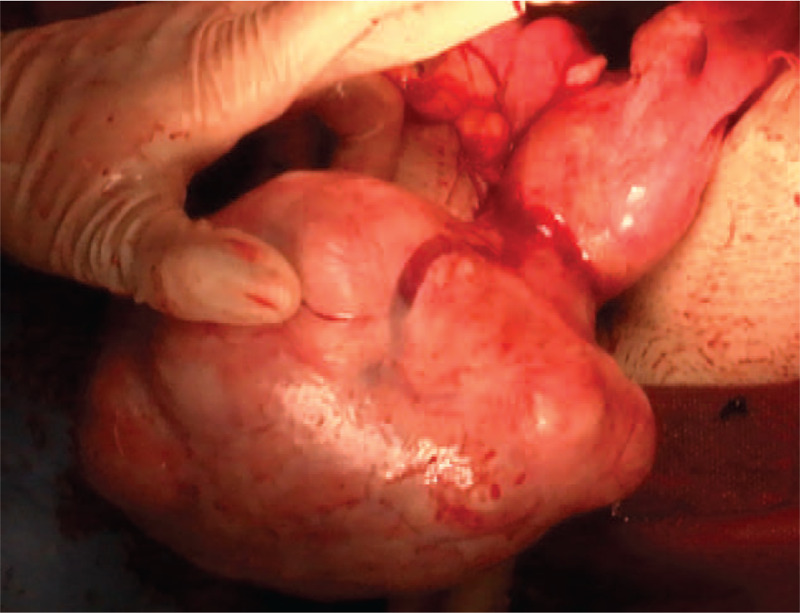
Active bleeding from the superficial ruptured vessel overlying the subserosal fibroid on the uterine fundus.

A 48-year-old woman with 2 gravida and her first menses at 11 years of age had a regular menstrual cycle with 4 to 5 days of bleeding each month. She had medical history of uterine fibroid and no remarkable family history. Her body mass index was 24 kg/m^2^. She was admitted to the emergency room complaining of acute-onset lower abdominal pain on the first day of her menstrual cycle. Her heart rate was 104 beats per minute, blood pressure was 81/51 mm Hg, respiratory rate was 20 per minute, and temperature was 35.8° C. Examination revealed generalized tenderness and a positive Blumberg sign. Transabdominal ultrasonography (US) revealed hyperechoic fluid filling almost the entire abdominal cavity and a uterine fibroid with a diameter of 11 cm. The patient had a reduced hemoglobin level (5.6 g/dL) and hematocrit (19.4%). The urine pregnancy test was negative. Abdominal contrast-enhanced CT showed a large amount of hemorrhagic ascites, a uterine fibroid (diameter 11 cm) and extravasation from the dilated vessel on the dorsal side of the uterine fibroid (Fig. 1). Exploratory laparotomy was performed under preoperative diagnosis of hemoperitoneum associated with uterine fibroid. During laparotomy, a large amount of hemorrhagic ascites was noted along with subserosal uterine fibroid (diameter 11 cm). Active bleeding was occurring from superficial ruptured vessels overlying the subserosal fibroid on the uterine fundus (Fig. 2). Total abdominal hysterectomy and bilateral salpingectomy were performed. The total amount of bleeding was 4,600 mL. Intraoperative blood salvage (1,357 mL restored by Cell Saver; Haemonetics Co., Braintree, MA), 6 units of red blood cells, 4 units of fresh frozen plasma, and 20 units of platelet concentrates were transfused. The postoperative course was uneventful. Pathological examination confirmed the diagnosis of benign uterine fibroid.

## 3 Discussion

In the current case, extravasation from the dilated vessels of the uterine fibroid was confirmed using contrast-enhanced CT, which enabled a preoperative diagnosis.

In the literature, approximately 100 cases of hemoperitoneum resulting from uterine fibroids have been reported.^[5]^ We searched the literature using the term leiomyoma/fibroids and hemoperitoneum in the PubMed database and found 42 cases reported from February 1980 to July 2019. The most common cause of hemoperitoneum associated with uterine fibroids was rupture of the serosal vessels (26/42 cases). The mechanism that precipitates the rupture of serosal vessels in such cases is yet unclear. Several factors, including trauma, exercise, defecation, menstruation, and alcohol consumption have been reported as causing the rupture of serosal vessels overlying the subserosal uterine fibroid.^[6,7]^ In this report, there were 14 cases (53.8%) of unknown cause, 7 cases (26.9%) during menstruation, 3 cases (11.5%) during defecation, and 1 case (3.8%) of falls and exercise. For present case, we speculate that the increased congestion of the superficial veins of uterine fibroids owing to menstruation may have contributed to the venous rupture on the first day of menstruation.

Preoperative diagnosis was extremely difficult due to the rareness.^[6,8]^ Imaging techniques, such as US and CT, are commonly used for preoperative examination, but in most of cases preoperative diagnosis was unexplained hemoperitoneum. In fact, 73.1% (19/26) of cases in this survey were diagnosed with unexplained hemoperitoneum. To the best of our knowledge, there were no cases of preoperative diagnosis of hemoperitoneum due to bleeding from uterine fibroids. Since the emergency operation must performed as soon as possible, clinical examination with US is sufficient for preoperative diagnosis.^[9]^ However, when cause of hemoperitoneum is not derived from gynecological organs, the cooperation of gastroenterological or cardiovascular surgeons is required. Therefore, we consider that CT is helpful not only for preoperative diagnosis, but also to exclude other intra-abdominal and extragenital pathologies.

Treatment is usually surgery such as hysterectomy and myomectomy. The choice of procedure is hysterectomy for women who are postmenopausal or do not wish to have children and myomectomy for younger women or those who wish to preserve their fertility. In this report, myomectomy was performed in 64% of cases (16/25) and hysterectomy was performed in 36% of cases (9/25). Outcomes are generally good if the appropriate surgery is performed.

In this case, extravasation from the dilated vessels of the uterine fibroid was confirmed using contrast-enhanced CT, and it was possible to determine a preoperative diagnosis of hemoperitoneum with bleeding from uterine fibroids.

## Acknowledgments

We would like to thank Editage (www.editage.com) for English language editing.

## Author contributions

**Writing – original draft:** Atsushi Daimon, Tomohito Tanaka.

**Writing – review & editing:** Tomohito Tanaka, Yuhei Kogata, Yoshimichi Tanaka, Daisuke Fujita, Masahide Ohmichi.
